# Retraction: LncRNA NEAT1 enhances the radio-resistance of cervical cancer via miR-193b-3p/CCND1 axis

**DOI:** 10.18632/oncotarget.28416

**Published:** 2023-04-24

**Authors:** Dongmei Han, Jianfeng Wang, Guanghui Cheng

**Affiliations:** ^1^Department of Radiation Oncology, China-Japan Union Hospital of Jilin University, Changchun 130033, China

**This article has been retracted:** Several images in this paper are duplicates of images in other published papers. An image in panel B of Figure 8 is a duplicate of an image in panel C, Figure 2 of another published paper [1]. An image in panel C, Figure 3 is a duplicate of an image in panel C, Figure 2 of another published paper [2]. Although this article was published earlier than the other articles, the Oncotarget Scientific Integrity Office decided to retract this article given concerns about the reliability of the data. All authors have agreed to this retraction.

Original article: Oncotarget. 2018; 9:2395–2409. 2395-2409. https://doi.org/10.18632/oncotarget.23416
